# Delirium education and post-anaesthetics care unit nurses’ knowledge on recognising and managing delirium in older patients

**DOI:** 10.1007/s40520-023-02390-2

**Published:** 2023-04-04

**Authors:** Callum Ormonde, Ezinne O. Igwe, Jessica Nealon, Pauline O’Shaughnessy, Victoria Traynor

**Affiliations:** 1 Faculty of Science, Medicine and Health, School of Medicine, University of Wollongong, Wollongong, NSW Australia; 2 Faculty of Science, Medicine and Health, School of Nursing, University of Wollongong, Wollongong, NSW 2522 Australia; 3 Illawarra Health and Medical Research Institute (IHMRI), Wollongong, NSW Australia; 4 School of Mathematics and Applied Statistics, Faculty of Engineering and Information Sciences, University of Wollongong, Wollongong, Australia

**Keywords:** Postoperative delirium, Delirium education, Delirium risk factors, Post-anaesthesia care units, Registered nurses, Delirium care practice

## Abstract

**Background:**

Postoperative delirium (POD) is a major complication following a surgical procedure. There is evidence that improving knowledge about POD could enhance POD care and patient outcomes.

**Aim:**

The study aimed to evaluate whether the amount of delirium education among registered nurses working in post-anaesthetics care units (PACU) impacts on their self-reported confidence and competence in recognising and managing delirium as well as prior knowledge on factors that influence the risk of delirium onset for older people.

**Method:**

The current study utilised an online survey on delirium care practice among registered nurses in PACUs. The survey consisted of 27 items. There were questions about confidence and competence in delirium care, knowledge about delirium risk factors, and ranked responses to two case scenario questions to evaluate the application of POD care. There were also demographic questions, including previous experience with delirium care education.

**Results:**

A total of 336 responses were generated from registered nurses working in PACU. Our findings found substantial variability among the respondents about their delirium care education. The amount of delirium education did not influence the PACU registered nurses’ confidence or competence in delirium care. In addition, previous education did not have an impact on their knowledge about delirium risk factors.

**Discussion and conclusion:**

These findings suggested that the quantity of prior education about delirium did not improve the confidence, competence, knowledge, or case scenario questions of PACU registered nurses. Thus, delirium care education needs to be transformed to ensure it has a positive effect on delirium care clinical practice by registered nurses in PACU.

## Introduction

The rapid increase in the global aging population emphasises the need for enhanced aged care. Globally, the estimated population of people 65 years and over is 727 million and the number is expected to double by the year 2050 [1]. As such, improved hospital care for older population groups, including delirium care which causes a significant health burden is warranted [2]. Delirium is an acute, reversible disorder of attention and cognition [3]. Delirium can occur during hospital admission and when it occurs after a surgical procedure it is known as postoperative delirium (POD). POD is more common among older people [2] and is one of the major complications experienced by older people following a surgical procedure. There are evidence-based clinical practice guidelines for delirium management, however, the routine practice of delirium diagnosis in clinical settings differ widely [4–7]. This discrepancy in diagnostic criteria leads to inadequate delirium recognition, subsequently resulting in under-detection of delirium incidence, [5, 8] especially among older individuals. There is a need for a uniform approach in clinical practice relating to delirium. Delirium education is most effective when formal teaching is interactive and engages leadership in combined clinical pathways and evidence-based assessment tools [9, 10]. Fundamentally, more structured education sessions that emphasise evidence-based practices in multimodal/multiphase and incorporating objective structured clinical examination (OSCE) style education can empower nurses to feel they can effectively manage delirium in the workplace as well as maintain their mental health and feel safe [10–12].

The impact of POD on older people cuts across all aspects of healthcare including prolonged length of stay in the hospital, falls, mortality and repeat delirium as well as increased medical expense, higher rates of readmission and/or relocation to a nursing home after discharge [6, 7, 13, 14]. An episode of POD also adds the risk of developing cognitive impairment in more than 50% of surgical patients and this risk persist for one-year post-surgery [15–17]. Improving delirium care is imperative to address the healthcare burden of delirium and improve post-operative outcomes for older people. One way of achieving this is undertaking research that supports knowledge translation of evidence-based delirium care with registered nurses. One group of registered nurses where there is great potential to improve the implementation of evidence-based delirium care is those working in PACUs as PACUs are critical care units with close observation and the first point of recovery for surgical patients.

Education is the first stage of knowledge translation, as such, it is imperative that the knowledge of clinicians is first established before undertaking knowledge translation research. This study was undertaken as a part of knowledge translation research evaluating the impact of an education intervention on registered nurses working in PACUs, specifically improved confidence and competence in delirium care as well as detecting delirium in PACUs. It is valuable to understand whether the amount of delirium education among registered nurses, whether it be undergraduate, postgraduate, and/ or ongoing professional development experience, impacts on delirium care confidence, competence, and knowledge about recognising and managing POD in older individuals [18, 19].

Our research group at have an established track record in undertaking knowledge translation in the area of delirium care [20, 21] using Pathmas’s 4A model of knowledge translation [22, 23]. The delirium care pathway developed by our research team progressed into becoming the Australian Commission on Safety and Quality in Health Care delirium care national standard [24]. Following the pathway publication, the research team implemented evidence-based delirium care with industry partners through knowledge translation research including local policy development and implementation of education programmes. However, the under-detection of delirium remains a problem [25]. Our research team explored in more depth the impact of education on the confidence, competence, and knowledge among registered nurses in recognising and managing delirium in older people. The team developed a survey aimed at assessing the relationship between the amount of delirium education undertaken and self-reported confidence and competence in delirium care as well as preferences on factors that influence the risk of delirium. Evidence from this study will describe the current levels of confidence and competence about delirium care as well as knowledge about delirium risk factors and management among PACU registered nurses in Australia. It is imperative that registered nurses working in PACU have the appropriate knowledge and skills to deliver evidence-based prevention, recognition, and management of post-surgical complications, including delirium [10, 26]. Once this is established, knowledge translation research can be designed that addresses the gaps in the delivery of evidence-based delirium care in PACU.

## Methods

The study was approved by The University of Wollongong Social Sciences Human Research Ethics Committee (HREC) in compliance with the *National Statement on Ethical Conduct in Human Research 2007* (HREC 2019/PID00838).

The current study utilised an online survey for data collection. This survey was constructed using SurveyMonkey^®^ and distributed by email invitation to relevant nursing professional organisations in Australia. There was a total of 27 questions in the survey. Questions were adapted from similar surveys on the topic [27, 32] and were categorised into five groups:

- demographics;
- educational and professional background;
- workplace practices and protocols in relation to delirium care in older people;
- self-reported confidence and competence in recognising and managing delirium as well as prior knowledge on factors that influence the risk of delirium onset for older people; and
- clinical case scenario-focused questions about POD (Table 1).

**Table 1:** Demographics and characteristics of respondents

Demographic of respondents	*N* = 336 (%)
*Gender*	
Male Female Non-binary Prefer to self-describe Would prefer not to say	40 (11.9) 290 (86.3) 1 (0.3) 1 (0.3) 4 (1.2)
Age (mean ± SD)	47 ± 2
*Position*	
Certified registered nurse anaesthetist CRNA/ Clinical nurse consultant (CNC) Clinical nurse educator (CNE) Nurse unit manager (NUM) Clinical nurse specialist (CNS) Registered nurse (RN)	18 (5.4) 25 (7.4) 47 (14) 75 (22.3) 153 (45.5)
*Highest academic qualification*	
Hospital qualification/diploma/certificate Bachelor’s degree Graduate diploma Masters and PhD degree	42 (12.5) 114 (33.9) 120 (35.7) 59 (17.6)
*Level of delirium care education*	
No education activities 1–2 education activities 3 education activities	102 (30.4) 213 (63.4) 20 (6.0)

The questions about delirium education asked respondents to report on their undergraduate, postgraduate, and ongoing professional development experiences related to delirium. The scenario questions asked respondents to report how they would clinically respond to the delirium cases presented. Response formats to the questions were determined by the type of question asked and comprised of Likert scale items and fixed category responses.

Respondents self-reported their level of confidence and competence in four aspects of POD care:

(i) recognising hyperactive delirium (characterised by increased motor activity, restlessness, agitation, aggression, wandering, hyper-alertness, hallucinations and delusions, and inappropriate behaviour);
(ii) recognising hypoactive delirium (characterised by reduced motor activity, lethargy, withdrawal, drowsiness and staring into space);
(iii) managing hyperactive delirium; and
(iv) managing hypoactive delirium.

Confidence and competence levels were rated on a four-point Likert scale as ‘no confidence/competence,’ ‘slight confidence/competence,’ ‘moderate confidence/competence’ or ‘high confidence/competence.’

### Validity and reliability of survey

Validity of the survey questions was undertaken in a range of ways. Content validity of the survey questions was reviewed by the whole research team as well as an independent panel consisting of seven researchers and clinicians with expertise in delirium care representing different multi-disciplinary team members comprising of an anaesthetist, a medical scientist, three nurses, a pharmacist, and a public health specialist. This review process was an iterative process with several rounds of comments sent back and forth. Edits and modifications, including deleting questions continued until they reached a consensus about the survey questions.

Following the content validity, face validity was completed with ten registered nurses working in a local PACU who accepted an invitation to participate in a face-to-face focus group to undertake a ‘read aloud’ activity with a member of the research team. During the ‘read aloud’ activity, the registered nurses provided feedback on the questions to ensure the intended meaning of each question was accurate. The feedback was incorporated to create the draft version of the survey.

Internal consistency testing of the survey questions was undertaken to determine the reliability of the survey. A draft version of the survey was pretested with 21 registered nurses working in a local PACU. Their responses were used solely for internal consistency testing and not included in the analysis for this study. The reliability of the survey questions was measured by checking using Cronbach’s Alpha whereby values above 0.7 are considered as acceptable [28]. The mean score for all survey questions in the current study instrument was 0.72. No further edits were made to the survey questions following the internal validity testing. The reliability of the survey was determined to be ‘acceptable’ and thus a definitive final version of the survey was ready for distribution.

### Recruitment

The study population was defined as registered nurses in Australia who worked as preoperative nurses, certified registered nurse anaesthetists, post anaesthesia care unit (PACU) nurses or recovery nurses. A snowballing technique was adopted to recruit respondents from across Australia. The research team drew on their collaborations and contacted professional organisations (*n* = 4) and hospitals (*n* = 3) to facilitate the distribution of a survey email invitation. A survey email invitation was sent to primary contacts asking that the email invitation be forwarded to other potential respondents who were invited to also forward the survey email invitation. Calculating a response rate was not possible. However, we concluded that the survey sample was representative of the Australian registered nurse population by comparing the characteristics of the respondents with nurses registered with the Australian Health Practitioner Regulation Authority (AHPRA). According to the 2021 Health Workforce Data [29], 11.6% of registered nurses are male in Australia while we recruited 11.9% of male respondents for this survey. The number of registered nurses who reported that they worked in the major cities was also comparable (population 72.7% vs sample 69.6%). Registered nurses aged 34 and under accounted for 31.9% of the registered nursing population, compared to 35% in the survey sample.

### Data analysis

Data were analysed using IBM SPSS Statistics for Windows, Version 21.0. (IBM Corp., Armonk, NY, USA). Frequencies and descriptive statistics for the demographic characteristics of the respondents were performed. Normality of continuous variables was assessed using Shapiro–Wilk tests. A one-way ANOVA (parametric) and Kruskal Wallis-*H* tests (non-parametric) were used to examine differences between groups for numeric data. Chi-square was used to determine the association between categorical variables about delirium care, such as confidence and competence in recognising and managing POD, knowledge about risk factors and clinical practice. Freidman test was used to analyse ranked data. All data were expressed as percentages or mean ± standard deviation (SD). Significance level was set at 5%.

## Results

### Respondent demographics

For the final analysis, a total of 336 respondents who answered the survey in part or completely were used. The study population was defined as registered nurses working in PACUs in Australia.

The survey respondents were categorised into three groups. These groups were based on the respondents’ answers to their delirium education during their undergraduate and postgraduate education as well as their ongoing professional development. The three groups are described thus:

- Group 1: completed no formal delirium care education—undergraduate, postgraduate, nor ongoing professional development (*n* = 102);
- Group 2: completed delirium care education from one or two of the described education categories—undergraduate, postgraduate, or ongoing professional development (*n* = 213); and
- Group 3: completed delirium care education in all three or more education categories (*n* = 20).

These three groups were used to explore the association between the level of delirium training and the ability of nurses to recognise and manage delirium in older patients.

Demographic and clinical practice characteristics of respondents were reported as means (SD) and proportions (%) (Table 1). The mean age of respondents was 47 years (± 2 years). Most of the respondents were female (86%). There was a higher proportion of respondents who identified as registered nurses (RN) (45%), followed by clinical nurse specialists (CNS) (22%) and nurse unit managers (NUM) (14%). Approximately two-thirds of respondents reported that their highest qualification was a graduate diploma (35%), or bachelor’s degree (33%) and a low proportion had master's or PhD degrees (17%).

### Respondent confidence and competence in the recognition and management of hyperactive and hypoactive delirium

**Fig. 1:**
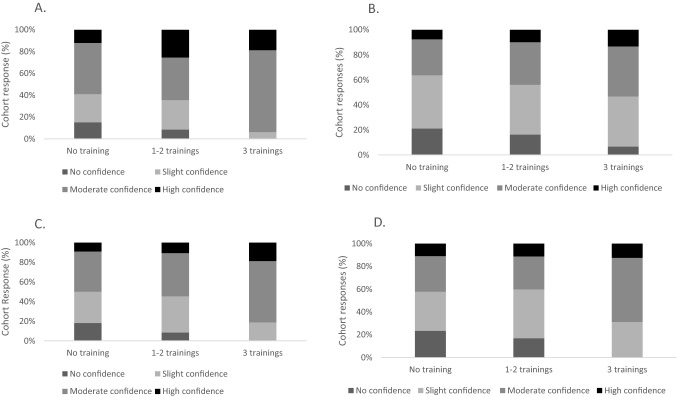
Respondent self-reported level of confidence, according to delirium education in** a** recognising hyperactive delirium, **b** recognising hypoactive delirium, **c** managing hyperactive delirium, **d** in managing hypoactive delirium

Figure 1 shows the levels of confidence in recognising and managing hyperactive and hypoactive delirium across three levels of delirium education among the respondents. In the four aspects of delirium care, most respondents within the groups ranked themselves as slightly or moderately confident (65–80%), with no confidence or high confidence in the minority of responses (0–20%). Each of the three groups reported more confidence in recognising (hyper/hypoactive) delirium (Fig. 1A, B) than in managing hyper- or hypo-active delirium (Fig. 1C, D). There was a statistically significant relationship between levels of delirium education and confidence in recognising hyperactive delirium ($\chi$^2^ = 17.634, *p* = 0.024, Fig. 1A) between the respondents.

**Fig. 2:**
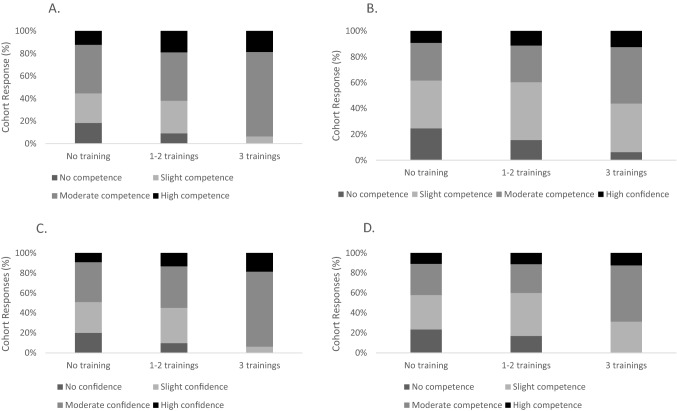
Respondent self-reported level of competence, according to delirium education, in **a** recognising hyperactive delirium, **b** recognising hypoactive delirium, **c** managing hyperactive delirium, **d** in managing hypoactive delirium

Figure 2 shows the pooled self-reported competence levels across these areas of delirium care education. Similar levels of confidence between the respondents, all groups identified the highest competence in recognising hyperactive delirium. There was a statistically significant association levels of delirium care education and their competence between the respondents in: (i) recognising hyperactive delirium (*p* = 0.048, Fig. 2A), and (ii) managing hyperactive delirium (*p* = 0.022 Fig. 2C).

Respondents were categorised into three groups based on their current positions (specialist nurse and above, unit manager, and registered nurse). No association was observed between position and the number of delirium training undertaken across the three groups (*p* = 0.273) or competence/confidence levels.

### Respondent knowledge of risk factors

To determine the level of knowledge about delirium risk factors a list of 18 possible perioperative risk factors, identified from current literature, was presented to respondents. They were required to state whether each listed risk factor was either “new knowledge to me” or they were “already aware of this risk factor.” As shown in Fig. 5 respondents with no formal delirium care education (Group 1) had a mean score of 11.89 (± 3.81, range 0–18) out of the 18 listed risk factors. The group with one or two formal delirium care education (Group 2) scored a mean of 12.10 (± 3.26, range 3–18) and the group with three formal delirium care education had a mean score of 13.56 (± 3.43, range 8–18).

### Respondent care of a patient with postoperative delirium—case scenario 1

Respondents were presented with the case scenario in Table 2. This case scenario represents a typical patient with hyperactive delirium. Two questions asked respondents about the management of the delirium care case scenario. The first question asked respondents to prioritise the listed actions in the order they would complete in the management of POD in an older person. The four available responses (in correct ascending order) were: (1) reassure and reorient the patient, (2) evaluate the current environment, (3) administer pain relief, and (4) administer sedation. Highest priority answers were ranked as 1, with the lowest priority answer ranked as 4. A Freidman test was used to analyse ranked data according to the three categories. This result was used to determine how each group prioritised the possible interventions given in the scenario. Participants’ ranked responses are presented in Table 3.

**Table 2:** Case scenario presented to respondents

Case scenario 1:	
“An 82-year-old sustains a hip fracture and undergoes an emergency surgical procedure. Preoperative oxygen saturation is 88%, blood pressure, 160/100 and pulse 110. She has a fever, is agitated, and confused and has difficulties giving clear answers to questions asked of her. Her postoperative pain is relieved with opioids. She develops postoperative delirium.”

**Table 3:** Ranked responses to question 1—case scenario 1

Amount of delirium education	1st priority	2nd priority	3rd priority	4th priority
Group 1 (no education)	Reassure and reorient	Administer sedation	Administer pain relief	Evaluate current environment
Group 2 (1–2 education sessions)	Administer pain relief	Evaluate current environment	Administer sedation	Reassure and reorient
Group 3 (3 education sessions)	Reassure and reorient	Evaluate current environment	Administer pain relief	Administer sedation
Ideal response	Reassure and reorient	Evaluate current environment	Administer pain relief	Administer sedation

In the second delirium care case scenario question, respondents were asked to rank management options based on a scenario where the older person’ POD persisted for more than one hour. The four options provided were: (1) Request urgent medical review, (2) Request to administer antipsychotics, (3) Request to increase the level of sedation, and (4) Apply restraints to the patient. Participants’ ranked responses are presented in Table 4.

**Table 4:** Ranked responses to question 2—case scenario 1

Amount of delirium education	1st priority	2nd priority	3rd priority	4th priority
Group 1 (no education)	Apply restraints	Request antipsychotics	Increase sedation	Medical review
Group 2 (1–2 education sessions)	Increase sedation	Request antipsychotics	Medical review	Apply restraints
Group 3 (3 education sessions)	Apply restraints	Request antipsychotics	Medical review	Increase sedation
Ideal response	Medical review	Increase sedation↔	Request antipsychotics	Apply restraints

## Discussion

### Overview

Findings from this study showed an association between increased delirium education and self-reported confidence and competence in recognising and managing hyperactive delirium among registered nurses. Current evidence shows that targeted educational interventions are successful in knowledge translation in clinical practice relating to delirium among registered nurses [30]. The impact of delirium education on clinical care knowledge for hospitalised people is inconsistent [12, 31], with nurses’ recognition of delirium ranging between 26 and 83% [32]. One meta-analysis found that educational intervention was independently effective in promoting clinicians’ adherence to guidelines [33]. This highlights the need for a consensus on guidelines and continuing professional development education about the recognition and management of delirium. It is important to highlight that the big variability in the study data may have accounted for non-significant results in some of the tests utilised.

### Respondents’ confidence and competence

Respondents in this study reported lower familiarity with hypoactive delirium. This observation provides insight into clinical practices and knowledge surrounding the recognition and management of delirium, specifically hypoactive delirium. Previous studies reported that a lack of knowledge and education reduced clinical confidence [34, 35]. One way to extend learning opportunities on specialist clinical topics like delirium care is to supplement these with workplace learning and peer support to improve the confidence and competence of nurses in areas where formal education is lacking [36, 37]. Registered nurses with higher levels of delirium education were significantly more likely to report higher confidence levels in recognising hyperactive or ‘typical’ delirium (*p* = 0.024). The presentation of hyperactive delirium is usually obvious, including agitation, hyper-arousal, loss of orientation, and language disturbance. Furthermore, the onset of hyperactive delirium presents challenges relating to physical and psychological exhaustion for nursing staff [38] with subsequent workplace education and support pertinent to staff competence and confidence. On the contrary, the more subtle nature of hypoactive delirium compared to the overt presentation of hyperactive delirium is a plausible explanation for this difference in confidence in recognising delirium subtypes [3, 39, 40]. Similarly, self-perception of competence in recognising hyperactive delirium among registered nurses was significantly associated with increased delirium care education. This result supports findings in the literature that competence is a process of knowledge and confidence [40–42]. Experience also contributes crucially to developing a sense of competence in the delivery of effective clinical care [42]. Considering the observed frequency of hypoactive delirium is substantially smaller compared to hyperactive delirium [3, 39, 40], registered nurses might perceive themselves less competent in the care of hypoactive delirium simply because of a lack of exposure to this sub-type of delirium.

### Case scenario and interventions

A recent systematic review [43] found that while nurses do maintain an in-depth theoretical knowledge of delirium, this knowledge does not always translate to adequate prevention, recognition, and management of delirium. Non-pharmacological treatments are the most effective evidence-based management for POD [44], however, results from this study showed that, on average, pharmacologic interventions were the top two priorities for participants. Additionally, nurses may be following cues learned from peers that do not follow evidence-based guidelines, as nurses commonly learn from peer practice to supplement areas where they are lacking in knowledge [34, 45]. A 2017 study of the experiences and perceptions of care nurses found they were sceptical about assessment tools, distrusted management protocols and found that the unpleasant nature of delirium was a pervasive belief held by nurses. These negative beliefs were said to impact practice and decision making [46]. Conversely, peer collaboration may be a preferred way of learning among nurses because it creates more supportive workplaces. This was especially true when speaking about patients with delirium who were described as patients who caused stress and were taxing [34]. The unpredictable nature of delirium can create concerns for safety, and this is one of Thomas et al.’s (2021) four themes that were described as influencing the nursing care of patients with delirium [45]. However, it is also possible that knowledge of evidence-based delirium care is lacking, and nurses are unaware of the correct management protocols. Clear delirium management protocols must be developed and implemented in PACU, and complimentary education sessions provided when new protocols are introduced to reinforce evidence-based delirium care.

### Strengths and limitation

Strengths in this study are seen in a large sample size of varied clinical experience and educational backgrounds. To replicate the current study, the survey would need to be adapted to other clinical settings or countries. Similar to how the survey was developed (from a Swedish study [27]), the scenario questions would need to be edited and amended to reflect clinical practice in different settings.

There is a possibility that the type of training received as well as repetitive training exposure may impact competences. The current study assessed respondents’ quantity of delirium education without evaluation of the delirium education content or frequency of delirium trainings. This limits the study outcomes, however, future studies could analyse delirium education content as well as frequency to understand how this may contribute to clinical practice in relation to delirium. Additionally, survey distribution for this study was restricted to perioperative and registered nurses working in PACUs Australia. Therefore, limitations must be made to the widespread application of findings to other ward practices or non-POD such as delirium tremens or withdrawal delirium. Results should also be interpreted with caution when considering survey answers are subject to self-reporting bias and the voluntary sample could represent respondents more motivated to learn than the greater nursing population. Although some evidence for the validity of self-rated competences has been described [47], undertaking audits of clinical records and observation of clinical practice is a proven method to determine whether levels of self-efficacy match clinical practices [48].

## Conclusion

This paper contributes to the understanding of the behaviours of registered nurses care for older patients with POD. Most registered nurses report themselves slightly moderately skilled at caring for patients with hyperactive or hypoactive delirium. Enhanced delirium education can improve some, but not all aspects of registered nurses’ confidence and perceived competence in delirium care. Prior education was an unreliable predictor of registered nurses’ behaviour in a clinical case scenario. Current directives to treat POD with non-pharmacological supportive means may be best for the patient outcomes but also create adverse outcomes for registered nurses’ stress level, confidence, as well as mental and physical health. Further research is needed to optimise the interplay of patient and professional outcomes. Focus should be given to how registered nurses cope with these challenges and how best to build their knowledge and confidence in caring for patients with POD. Future research could explore the geographical differences in this area. In addition, the expansion of multimodal, multiphase peer-support programs represents an acceptable strategy to improve safety, confidence, and competency in perioperative registered nurse practices.

## Data Availability

The authors declared data availability during submission. Due to ethics approval, there are restrictions and data sharing.
